# Dengue-2 and Guadeloupe Mosquito Virus RNA Detected in *Aedes* (*Stegomyia*) spp. Collected in a Vehicle Impound Yard in Santo André, SP, Brazil

**DOI:** 10.3390/insects12030248

**Published:** 2021-03-16

**Authors:** Marina E. O. Rangel, Luana P. R. Oliveira, Aline D. Cabral, Katharyna C. Gois, Marcos V. M. Lima, Beatriz C. A. A. Reis, Fernando L. A. Fonseca, Marcia A. Sperança, Flavia S. Gehrke, Gabriel Z. Laporta

**Affiliations:** 1 Setor de Pós-Graduação, Pesquisa e Inovação, Centro Universitário Saúde ABC (FMABC), Fundação ABC, Santo André 09060-870, Brazil; marina.rangel.bio@gmail.com (M.E.O.R.); marcos.malveira@ac.gov.br (M.V.M.L.); 2 Centro de Ciências Naturais e Humanas, Universidade Federal do ABC (UFABC), São Bernardo do Campo 09606-045, Brazil; luanaprolim@gmail.com (L.P.R.O.); alinedica@gmail.com (A.D.C.); marcia.speranca@ufabc.edu.br (M.A.S.); 3 Laboratório de Análises Clínicas, Centro Universitário Saúde ABC (FMABC), Fundação ABC, Santo André 09060-870, Brazil; katharyna.gois@outlook.com (K.C.G.); bcaalves@uol.com.br (B.C.A.A.R.); profferfonseca@gmail.com (F.L.A.F.); 4 Departamento de Farmácia, Universidade Federal de São Paulo (UNIFESP), Diadema 09972-270, Brazil; 5 Departamento de Patologia, Centro Universitário Saúde ABC (FMABC), Fundação ABC, Santo André 09060-870, Brazil; flaviagehrke@hotmail.com; 6 Programa de Pós-Graduação em Ciências da Saúde, Hospital do Servidor Público Estadual (IAMSPE), São Paulo 04039-000, Brazil; 7 Departamento de Farmácia, Universidade Paulista (UNIP), São Paulo 01504-001, Brazil

**Keywords:** arbovirus, mosquito-borne diseases, surveillance

## Abstract

**Simple Summary:**

We screened *Aedes* mosquitoes in an urban area in southeastern Brazil for emerging arboviruses using RT-PCR assays and sequencing. We detected DENV-2 RNA in *Aedes albopictus* and Guadeloupe mosquito virus RNA in *Aedes aegypti*.

**Abstract:**

In 2018–2019, we conducted mosquito collections in a municipal vehicle impound yard, which is 10 km from the Serra do Mar Environmental Protection Area in Santo André, SP, Brazil. Our aim is to study arboviruses in the impound yard, to understand the transmission of arboviruses in an urban environment in Brazil. We captured the mosquitoes using human-landing catches and processed them for arbovirus detection by conventional and quantitative RT-PCR assays. We captured two mosquito species, *Aedes aegypti* (73 total specimens; 18 females and 55 males) and *Ae. albopictus* (34 specimens; 27 females and 7 males). The minimum infection rate for DENV-2 was 11.5 per 1000 (CI95%: 1–33.9). The detection of DENV-2 RNA in an *Ae. albopictus* female suggests that this virus might occur in high infection rates in the sampled mosquito population and is endemic in the urban areas of Santo André. In addition, Guadeloupe mosquito virus RNA was detected in an *Ae. aegypti* female. To our knowledge, this was the first detection of the Guadeloupe mosquito virus in Brazil.

## 1. Introduction

The transmission dynamics of arboviruses of public health importance are currently not well understood in urban areas of Brazil [1,2,3]. Arboviral outbreaks periodically occur, causing widespread morbidity and mortality in human populations and other vertebrates [4,5,6]. Accumulated deforestation is a major driver of arbovirus spillover [7,8,9]. Human land-use activities bring humans into close contact with the sylvatic vectors, which may lead to spillover events in both humans and domestic animals [10,11]. This was what public health authorities were afraid of during the yellow fever (YF) outbreak, which lasted from 2016 to 2019 in Brazil [12]. This outbreak was caused by mutant strains of yellow fever virus (YFV) (Family *Flaviviridae*, Genus *Flavivirus*) [13]. These strains are highly virulent and caused death in 750 humans and thousands of new world monkeys [13]. Fortunately, *Aedes* (*Stegomyia*) *aegypti* (L.) has not shown competence to transmit these strains, which would have led to urban YF [14].

The danger of urban YF has been highlighted by the recent outbreaks of Zika virus (ZIKV) and chikungunya virus (CHIKV) [15,16]. There is currently an increased potential for disseminating YFV and other arboviruses of public health importance due to urban crowding conditions, rapid national and international air travel, and favorable climatic conditions [17,18,19]. The manifestation of clinical outcomes such as the newborn microcephaly associated with zika infections in pregnant women [20] further highlights the public health importance of *Ae. aegypti*, which is the primary vector for ZIKV, CHIKV, and dengue virus (DENV) [17]. The closely related *Aedes* (*Stegomyia*) *albopictus* (Skuse) is the primary vector of DENV in the United States [21] and in China [22] and of CHIKV in Europe [21], but has not been implicated as a vector in arboviral outbreaks in Brazil, so far [5].

Habitats for immature stages of *Ae. aegypti* and *Ae. albopictus* are artificial recipients in urbanized area of Santo André county, São Paulo state [23], where 3,356 dengue cases were confirmed from 2014 to 2017 [23]. Three YF human cases and four YF epizootics with dead monkeys were reported in 2018 [23]. Human cases caused by ZIKV and CHIKV infections have not been detected, so far. Because molecular surveillance techniques are not routinely used for arbovirus detection in mosquito vectors [24,25], ZIKV and CHIKV could be circulating undetected.

The success of the current arbovirus surveillance and control programs in Brazilian cities is limited by the limited availability of funds [18]. This problem is further compounded by a lack of specific therapeutics and the paucity of the licensed vaccines for most arboviral diseases [17]. It is increasingly important to develop novel vector surveillance and control strategies, with the primary goal of preventing spillover events initiating large-scale outbreaks [12]. One such strategy is to initiate and sustain molecular surveillance of RNA arboviruses in field-collected *Ae. aegypti* and *Ae. albopictus* populations, in order to effectively monitor potential vector-borne arboviral outbreaks in the future [14,24,25]. Arboviral molecular detection in mosquitoes relies on the use of reverse transcription (RT) PCR-based techniques, which are preferable to time-consuming in vitro cell culturing and more sensitive than serological testing [11,26,27]. The Sanger sequencing approach can quickly yield genetic sequences for arboviruses [28], which can be compared with the known sequences in GenBank [29]. Such comparison provides a powerful bioinformatics tool for quickly identifying the arboviral agents detected in field-collected mosquito vectors [28,30]. In this manuscript, we used the molecular surveillance technique to screen for arboviruses in mosquitoes collected in a vehicle impound yard, Santo André, SP, Brazil, from August 2018 to July 2019.

## 2. Materials and Methods

### 2.1. Study Area

**Figure 1:**
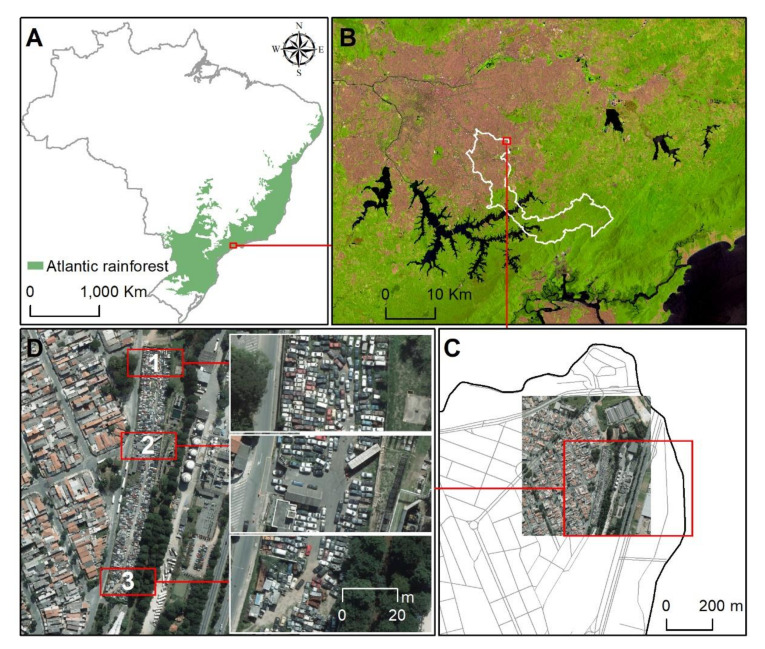
Study area: (**A**) Brazilian Atlantic rainforest; (**B**) municipality of Santo André (white borders) in the metropolitan region of São Paulo, state of São Paulo, southeastern Brazil; (**C**) vehicle impound yard in the Ana Maria Garden district; and (**D**) 1—3 are the sites where we collected mosquitoes. Sources: IBGE (biomes), USGS (Landsat 8OLI), SEMASA (aerophotogrammetric image), and ArcGIS v. 10.3.1.

Santo André is an industrial municipality within the metropolitan region of São Paulo, state of São Paulo, southeastern Brazil. More than 700 thousand people live in this municipality, and half of its 175 km^2^ territory is covered by the Atlantic rainforest (Figure 1A,B). DENV has been endemic to this area since the 2000s [23]; in addition, CHIKV, ZIKV, and YFV have recently been detected in the region [4,31,32].

The study area was a vehicle impound yard (−46.49 W, −23.63 S; see Figure 1C,D). The vehicle impound yard was an ideal location to monitor *Aedes* infestation. Impounded vehicles provide a wide variety of larval habitats, as well as resting sites for adult mosquitoes (Figure 1D).

### 2.2. Field Collections

Human-landing catches with small battery-powered aspirators were conducted biweekly for a one-year period from August 2018 to July 2019. Two catchers were used on each sampling occasion; the collections started at 9AM and ended at 1PM. The catchers wore trousers and long socks to minimize contact with wild mosquitoes [33].

The sampling sites were rotated every two weeks to eliminate collection site bias. All specimens (male or female) were transported live to the laboratory, chilled in a −20 °C freezer for 5 min, morphologically identified to species using the keys of Forattini [34], sorted by sex and collection date, and individually stored in microtubes in a −80 °C freezer.

### 2.3. RNA Extraction

The single-insect non-destructive RNA extraction method was used, following Molina et al. [28]. With this method, chitinous exoskeleton is preserved intact and the proteinaceous tissues are dissolved with proteinase K. The RNA can then be extracted from the solution [35]. To each individual mosquito, 200 uL of lysis buffer [200 mM Tris HCl (pH 7.5), 250 mM NaCl, 25 mM EDTA 372 (pH 8.0), 0.5% SDS, and 400 μg/mL proteinase K] were added, following incubation for 16 h at 56 °C. Total RNA was purified by using Invitrogen PureLink RNA Mini Kits (ThermoFisher Scientific, Waltham, MA, USA), following to the manufacturer’s instructions, and eluted in 60 uL of RNase free water. The concentration of the extracted RNA was evaluated by spectrophotometry using BioDrop μLITE (Biochrom, Harvard Bioscience, Holliston, MA, USA).

### 2.4. Arbovirus Detection

To screen the mosquitoes for arboviruses, cDNA from 5.5 μL total RNA of each specimen was synthesized using the MMLV reverse transcriptase purchased from ThermoFisher Scientific (Waltham, MA, USA) according to manufacturer’s instructions, using two different approaches: one with specific arboviruses oligonucleotides and the other with random hexamers. The reverse transcription reactions were set up at 42 °C for one hour, followed by enzyme inactivation at 80 °C for 5 min.

The cDNA synthesized with specific oligonucleotides were used to detect arboviruses by endpoint PCR in order to detect flaviviruses, South American alphaviruses, and CHIKV, according to methods previously described [28,36,37,38,39]. The primer sequences, cycling condition, target regions, and expected DNA fragment size we used are outlined in Table S1. PCR was performed with Platinum Taq DNA polymerase purchased from ThermoFisher Scientific according to manufacturer’s instructions and the obtained products were subjected to 1.5% agarose gel electrophoresis in 0.5 × TBE buffers after staining with SafeBlue. All PCR fragments with the expected size were ligated into pGEM-T vector (Promega)—transformed into Mach1-T1^R^ chemical competent *Escherichia coli* strain (ThermoFisher Scientific). Ampicillin-resistant colonies were selected. From each amplicon, two to three recombinant plasmids were purified using the Thermo Scientific GeneJET plasmid Purification Kit (ThermoFisher Scientific, Waltham, MA, USA) and sequenced by Sanger methodology [28] with BigDye v3.1 (Applied Biosystems) and oligonucleotides M13F and M13R, complementary to vector sequence. Sequence cycle conditions were 96 °C for 1 min, 39 cycles at 96 °C for 15 s, 50 °C for 15 s, and 60 °C for 4 min. Sequencing reactions were loaded in an ABI PRISM 3130XL Genetic Analyzer/HITACHI (16 capillaries) (ThermoFisher Scientific, Waltham, MA, USA). The source and specificity of the obtained sequences were evaluated by BLAST in GenBank [29,30].

The cDNA synthesized with random hexamers was used to detect the arboviruses by three multiplex real time PCR (RT-qPCR) reactions according to the methodology approved by the Sociedade Brasileira de Patologia Clínica, division of Laboratorial Medicine and routinely used in the diagnosis of DENV serotypes 1–4, CHIKV, ZIKV, and YFV of human samples, in the Clinical Laboratory of the Faculdade de Medicina do ABC (Gehrke et al., unpublished data). The primers and probes used in the reactions are summarized in Table S2 and each reaction was divided into: multiplex 1 (DENV-1, DENV-2, and YFV); multiplex 2 (DENV-3 and DENV-4); and multiplex 3 (CHIKV and ZIKV). The RT-qPCR reactions were carried out in an ABI Prism 7500 Fast thermal cycler (Applied Biosystems, Foster City, CA, USA), using the RT-qPCR expression kit purchased from ThermoFisher Scientific, with an initial cycle of 95 °C for 3 min, followed by 45 cycles of 95 °C for 15 s and 60 °C for 30 s, in a final volume of 15 µL. To identify the positive and negative samples, positive and negative controls were included on each sample plate. Samples with Ct < 39 were considered positive. The positive controls were kindly provided by external partners (see Acknowledgments). Two uL of each pool of up to 10 cDNA samples were used in each reaction. The mosquito pools were set up by adding 0.3 μL of each individual sample—or 3 μL from 10 individual samples—and 27 μL of water, totaling 30 μL in the microtube. If a pool went positive in any testing, we opened it and tested each mosquito specimen individually, using the same approach.

The minimum infection rate (MIR) was calculated by the number of positive pools/total mosquitoes tested × 1000. Confidence intervals of 95% were calculated as the sample proportion ± 1.96 times the standard deviation of the sample proportion × 1000. If MIR > 0, the CI95% lower limit was assumed as 1.

## 3. Results and Discussion

The total human-landing catch sampling effort was 192 man-hours (12 months × 2 collections per month × 2 collectors × 4 h per collection). We captured two species: *Ae. aegypti*, 73 specimens (18♀, 55♂) and *Ae. albopictus*, 34 specimens (27♀ and 7♂). RNA extraction from individual mosquitoes was successful, resulting in a total of 107 samples for arbovirus screening testing.

### 3.1. Endpoint PCR

We tested all 107 samples in endpoint PCR, resulting in fragments of expected sizes after agarose gel electrophoresis. Considering results from these fragments, we could detect 14 *Flavivirus* NS5 encoding gene, 3 *Alphavirus* nonstructural protein 1 gene, and 6 CHIKV E2 gene in 21 mosquito specimens (Table 1). After cloning and sequencing, however, these amplicons resulted in unspecific sequences in BLAST analysis, except in one case.

Amplicons with expected size for *Flavivirus* NS5-encoding gene in 14 *Ae. aegypti* specimens (Table 1) resulted in unknown sequences or in sequences with similarity to *Ae. aegypti* glycogen debranching enzyme (GenBank: XM_021853942.1) or to *Ae. aegypti* unknown mRNA sequence (GenBank: AY433243.1). Amplicons with expected size for *Alphavirus* nonstructural protein 1 gene in one female *Ae. aegypti* and two males *Ae. albopictus* (Table 1) showed no similarity to known GenBank sequences.

Amplicons with expected size for CHIKV E2 gene were obtained from six specimens (Table 1). While amplicons of five specimens showed unspecific sequences in BLAST analysis, two clones of an amplicon obtained from a female of *Ae. aegypti* (Table 1; ID 2) were identified as an unclassified virus belonging to the *Riboviria* group. Nucleotide sequence of this amplicon (341 bp) corresponded to the bases from 64 to 387 of the gene ORF2, which encodes an RNA-dependent RNA polymerase. This nucleotide sequence was 98% like Guadeloupe mosquito virus (GenBank: MN053797). Its amino acid sequence was 99% to the amino acid sequence from 22 to 129, of the RNA-dependent RNA polymerase of Guadeloupe mosquito virus. The nucleotide sequence of the obtained cDNA fragment (amplicon 341 bp) was deposited in GenBank under the accession number MW414904.

Guadeloupe mosquito virus is a newly discovered virus [40]. This virus does not infect vertebrate cells; however, it has been suggested that the origin of human pathogenic viruses may be tied to the evolution of arthropod-specific viruses [41,42]. With the growing number of mosquito-specific viruses identified in recent years, one question that arises is how—if at all—these viruses can have a specific influence on the transmission of pathogenic arboviruses to humans and animals. The closest relatives to the newly identified Guadeloupe mosquito virus are *Wenzhou sobemo*-like virus-4 (WSLV4) and *Hubei* mosquito virus-2 (HMV2) [40]. Another interesting point is that this virus could also be used in phylogenetic studies of *Ae. aegypti*. The Euclidean distance from the first detection of this virus in Guadeloupe Island in the Caribbean [40] to our study site is 3000 miles. This virus may be widely distributed in *Ae. aegypti* populations, suggesting that genetic variance among this virus population could be associated with the genetic variation among *Ae. aegypti* populations in the Americas.

### 3.2. RT-qPCR Assay

Considering unconfirmed positivity detection in endpoint PCR (Table 1), we sought to further test them using specific primers for DENV-1–4, CHIKV, ZIKV, and YFV. A total of 87 out of 107 specimens were screened for arbovirus RNA (Table 1). One pool containing 8 *Ae. aegypti* females and 2 *Ae. albopictus* was positive for DENV-2 with a Ct < 39 (Table 1; ID 26–28, 30–32, 37, 39–41). This pool was opened, and specimens were tested individually. Female *Ae. albopictus* (ID 39) went positive for DENV-2 testing (Table 1), which showed reaction curve of Ct = 38.7 in amplification plot (Figure S1).

The minimum infection rate for DENV-2 was 11.5 ([1/87] × 1000; CI95%: 1–33.9), which corresponds to 1% of infected mosquitoes. This result suggests that DENV-2 is hyperendemic in the vehicle impound yard, in urban area of Santo André. Other studies have found comparable DENV minimum infection rates in other endemic areas in Brazil [43,44] and abroad [45,46]. The minimum infection rate for DENV has been estimated to be 19.8 per 1000, considering both *Ae. aegypti* and *Ae. albopictus*, in northeastern Brazil [43] and ranged between 0 and 5.5 per 1000 in *Ae. albopictus* in Indonesia [47]. The minimum infection rate (11.5/1000) for DENV-2 reported here is considerably high, but was not surprising because dengue cases have been detected every year in Santo André [6,23].

We did not detect YFV, ZIKV, and CHIKV in our samples. This result was not surprising either. Yellow fever virus has not been detected in *Ae. aegypti* in Brazil in the recent past including the re-emergence from 2016–2019 [13,14,31]. Thus, urban YF, YFV-transmitted by *Ae. aegypti*, is unlikely. ZIKV and CHIKV have spread from the northeast to all over Brazil [4,16,32], but the incidence in southern Brazil has been very low, particularly in São Paulo [48]. However, there is the possibility that these viruses are circulating but at exceptionally low levels and thus difficult to detect in small sample sizes. As with other methods, the sensitivity of molecular surveillance increases with sample size.

Testing of the *Ae. albopictus* specimen (Table 1, ID 39) went positive for DENV-2 when RT-qPCR was employed; however, it went negative when we applied endpoint PCR targeting *Flavivirus* NS5-encoding gene. This discrepancy can be explained by comparative studies that showed higher sensitivity of RT-qPCR to detect DENV than that of endpoint PCR [49,50,51].

## Figures and Tables

**Table 1:** Results of virus RNA testing of *Aedes* (*Stegomyia*) spp. in endpoint PCR and RT-qPCR assays.

	Specimens				Endpoint PCR			RT-qPCR ^1^		
ID	Sex	Species	Sample	Site	NS5	Chik1/4	M2W/cM3W	Multiplex 1	Multiplex 2	Multiplex 3
1	F	albopictus	1	1	-	-	-	ND	ND	ND
2	F	aegypti	2	1	X	GMV	-	-	-	-
3	F	albopictus	3	1	-	-	-	ND	ND	ND
4	M	aegypti	5	2	-	-	-	-	-	-
5	M	aegypti	6	2	-	X	-	-	-	-
6	F	aegypti	7	1	-	-	-	ND	ND	ND
7	F	albopictus	8	1	-	-	-	ND	ND	ND
8	F	albopictus	10	1	-	-	-	ND	ND	ND
9	F	albopictus	9	3	-	-	-	-	-	-
10	F	albopictus	9	3	-	-	-	-	-	-
11	M	aegypti	4	1	-	-	-	-	-	-
12	M	aegypti	4	1	-	-	-	-	-	-
13	M	aegypti	4	1	-	X	-	-	-	-
14	F	aegypti	11	1	X	-	-	-	-	-
15	F	aegypti	11	1	-	-	-	-	-	-
16	F	aegypti	11	1	X	-	X	-	-	-
17	F	aegypti	11	1	X	-	-	-	-	-
18	M	aegypti	12	1	-	-	-	-	-	-
19	M	aegypti	12	1	-	-	-	-	-	-
20	M	aegypti	12	1	-	-	-	-	-	-
21	M	aegypti	12	1	-	-	-	-	-	-
22	M	aegypti	12	1	-	-	-	-	-	-
23	M	aegypti	12	1	-	-	-	-	-	-
24	F	aegypti	23	2	X	-	-	-	-	-
25	F	aegypti	13	3	X	-	-	-	-	-
26	F	aegypti	13	3	X	-	-	-	-	-
27	F	aegypti	13	3	X	-	-	-	-	-
28	F	aegypti	13	3	X	-	-	-	-	-
29	F	aegypti	13	3	-	-	-	ND	ND	ND
30	F	aegypti	13	3	X	-	-	-	-	-
31	F	aegypti	13	3	X	-	-	-	-	-
32	F	aegypti	13	3	X	-	-	-	-	-
33	F	albopictus	14	3	-	-	-	ND	ND	ND
34	F	albopictus	14	3	-	-	-	ND	ND	ND
35	F	albopictus	14	3	-	-	-	ND	ND	ND
36	F	albopictus	14	3	-	-	-	ND	ND	ND
37	F	albopictus	14	3	-	-	-	-	-	-
38	F	albopictus	14	3	-	-	-	ND	ND	ND
39	F	albopictus	14	3	-	-	-	DENV-2	-	-
40	F	aegypti	21	3	X	-	-	-	-	-
41	F	aegypti	21	3	X	-	-	-	-	-
42	F	aegypti	21	3	-	-	-	ND	ND	ND
43	F	albopictus	22	3	-	-	-	ND	ND	ND
44	F	albopictus	22	3	-	-	-	ND	ND	ND
45	F	albopictus	22	3	-	-	-	ND	ND	ND
46	F	albopictus	22	3	-	-	-	ND	ND	ND
47	F	albopictus	22	3	-	-	-	-	-	-
48	F	albopictus	22	3	-	-	-	ND	ND	ND
49	M	aegypti	16	3	-	-	-	-	-	-
50	M	aegypti	16	3	-	-	-	ND	ND	ND
51	M	aegypti	16	3	-	-	-	-	-	-
52	M	aegypti	16	3	-	-	-	-	-	-
53	M	aegypti	16	3	-	-	-	-	-	-
54	M	aegypti	16	3	-	-	-	ND	ND	ND
55	M	aegypti	16	3	-	-	-	-	-	-
56	M	aegypti	16	3	-	-	-	-	-	-
57	M	aegypti	16	3	-	-	-	-	-	-
58	M	aegypti	16	3	-	-	-	ND	ND	ND
59	M	aegypti	16	3	-	-	-	-	-	-
60	M	aegypti	16	3	-	-	-	ND	ND	ND
61	M	aegypti	18	3	-	-	-	ND	ND	ND
62	M	aegypti	18	3	-	-	-	ND	ND	ND
63	M	aegypti	18	3	-	-	-	ND	ND	ND
64	M	aegypti	18	3	-	-	-	-	-	-
65	M	aegypti	18	3	-	-	-	ND	ND	ND
66	M	aegypti	18	3	-	-	-	-	-	-
67	M	aegypti	18	3	-	-	-	-	-	-
68	M	aegypti	18	3	-	-	-	ND	ND	ND
69	M	aegypti	18	3	-	-	-	-	-	-
70	M	aegypti	18	3	-	-	-	ND	ND	ND
71	M	aegypti	18	3	-	-	-	ND	ND	ND
72	M	aegypti	18	3	-	-	-	-	-	-
73	M	aegypti	15	3	-	-	-	ND	ND	ND
74	M	aegypti	15	3	-	-	-	-	-	-
75	M	aegypti	15	3	-	-	-	ND	ND	ND
76	M	aegypti	15	3	-	-	-	-	-	-
77	M	aegypti	15	3	-	-	-	ND	ND	ND
78	M	aegypti	15	3	-	-	-	-	-	-
79	M	aegypti	15	3	-	-	-	ND	ND	ND
80	M	aegypti	15	3	-	-	-	ND	ND	ND
81	M	aegypti	15	3	-	-	-	-	-	-
82	F	albopictus	17	3	-	-	-	-	-	-
83	F	albopictus	17	3	-	-	-	-	-	-
84	F	albopictus	17	3	-	-	-	-	-	-
85	F	albopictus	17	3	-	-	-	-	-	-
86	F	albopictus	17	3	-	-	-	-	-	-
87	F	albopictus	17	3	-	-	-	-	-	-
88	F	albopictus	17	3	-	-	-	ND	ND	ND
89	M	aegypti	24	2	-	-	-	-	-	-
90	M	aegypti	24	2	-	-	-	ND	ND	ND
91	M	aegypti	24	2	-	-	-	ND	ND	ND
92	M	aegypti	24	2	-	-	-	-	-	-
93	M	aegypti	19	3	-	-	-	-	-	-
94	M	aegypti	19	3	-	-	-	ND	ND	ND
95	M	aegypti	19	3	-	X	-	-	-	-
96	M	aegypti	19	3	-	-	-	-	-	-
97	M	aegypti	19	3	-	-	-	-	-	-
98	M	aegypti	19	3	-	-	-	ND	ND	ND
99	M	aegypti	19	3	-	-	-	-	-	-
100	M	aegypti	19	3	-	-	-	ND	ND	ND
101	M	albopictus	20	3	-	X	-	-	-	-
102	M	albopictus	20	3	-	-	X	-	-	-
103	M	albopictus	20	3	-	X	-	-	-	-
104	M	albopictus	20	3	-	-	-	-	-	-
105	M	albopictus	20	3	-	-	-	-	-	-
106	M	albopictus	20	3	-	-	-	-	-	-
107	M	albopictus	20	3	-	-	X	-	-	-

RT-qPCR^1^: *multiplex* *1*, DENV-1, DENV-2, and YFV; *multiplex* *2*, DENV-3 and DENV-4; *multiplex* *3*, CHIKV and ZIKV. GMV: Guadeloupe mosquito virus RNA detection; X: unspecific sequence detection; -: no detection; ND: not done.

## Data Availability

The data is available on the article and on its supplementary material.

## Supplementary Materials

The following are available online at https://www.mdpi.com/2075-4450/12/3/248/s1, Table S1, Oligonucleotides and target regions in endpoint PCR assay; Table S2, Oligonucleotides, probes, and targets in RT-qPCR assay; Figure S1, Reaction curve of *Ae. albopictus* female specimen (39) in amplification plot in RT-qPCR.

## References

[1] Barreto F.K.A., Alencar C.H., Araújo F.M.C., Oliveira R.M.A.B., Cavalcante J.W., Lemos D.R.Q., Farias L.A.B.G., Boriz I.L.F., Medeiros L.Q., Melo M.N.P. (2020). Seroprevalence, Spatial Dispersion and Factors Associated with Flavivirus and Chikungunya Infection in a Risk Area: A Population-Based Seroprevalence Study in Brazil. BMC Infect. Dis..

[2] Jesus M.C.S., Chagas R.D.O., Santos C.A., Santos R.W.F., Barros G.S., La Corte R., Batista M.V.A., Storti-Melo L.M. (2020). Molecular Characterization and Phylogenetic Analysis of Chikungunya Virus during the 2016 Outbreak in Sergipe, Northeastern Brazil. Trans. R. Soc. Trop. Med. Hyg..

[3] Carabali M., Harper S., Lima Neto A.S., Dos Santos de Sousa G., Caprara A., Restrepo B.N., Kaufman J.S. (2020). Spatiotemporal Distribution and Socioeconomic Disparities of Dengue, Chikungunya and Zika in Two Latin American Cities from 2007 to 2017. Trop. Med. Int. Health.

[4] Costa A.C., Thézé J., Komninakis S.C.V., Sanz-Duro R.L., Felinto M.R.L., Moura L.C.C., Barroso I.M.O., Santos L.E.C., Nunes M.A.L., Moura A.A. (2017). Spread of Chikungunya Virus East/Central/South African Genotype in Northeast Brazil. Emerg. Infect. Dis..

[5] Cunha M.S., Faria N.R., Caleiro G.S., Candido D.S., Hill S.C., Claro I.M., da Costa A.C., Nogueira J.S., Maeda A.Y., da Silva F.G. (2020). Genomic Evidence of Yellow Fever Virus in Aedes Scapularis, Southeastern Brazil, 2016. Acta Trop..

[6] De Lima M.V.M., Laporta G.Z. (2020). Evaluation of the Models for Forecasting Dengue in Brazil from 2000 to 2017: An Ecological Time-Series Study. Insects.

[7] Vittor A.Y., Armien B., Gonzalez P., Carrera J.-P., Dominguez C., Valderrama A., Glass G.E., Beltran D., Cisneros J., Wang E. (2016). Epidemiology of Emergent Madariaga Encephalitis in a Region with Endemic Venezuelan Equine Encephalitis: Initial Host Studies and Human Cross-Sectional Study in Darien, Panama. PLoS Negl. Trop. Dis..

[8] Loaiza J.R., Dutari L.C., Rovira J.R., Sanjur O.I., Laporta G.Z., Pecor J., Foley D.H., Eastwood G., Kramer L.D., Radtke M. (2017). Disturbance and Mosquito Diversity in the Lowland Tropical Rainforest of Central Panama. Sci. Rep..

[9] De Lima R.A.F., Oliveira A.A., Pitta G.R., de Gasper A.L., Vibrans A.C., Chave J., Ter Steege H., Prado P.I. (2020). The Erosion of Biodiversity and Biomass in the Atlantic Forest Biodiversity Hotspot. Nat. Commun..

[10] Johnson C.K., Hitchens P.L., Pandit P.S., Rushmore J., Evans T.S., Young C.C.W., Doyle M.M. (2020). Global Shifts in Mammalian Population Trends Reveal Key Predictors of Virus Spillover Risk. Proc. R. Soc. B.

[11] Kauffman E.B., Jones S.A., Dupuis A.P., Ngo K.A., Bernard K.A., Kramer L.D. (2003). Virus Detection Protocols for West Nile Virus in Vertebrate and Mosquito Specimens. J. Clin. Microbiol..

[12] De Oliveira Figueiredo P., Stoffella-Dutra A.G., Barbosa Costa G., Silva de Oliveira J., Dourado Amaral C., Duarte Santos J., Soares Rocha K.L., Araújo Júnior J.P., Lacerda Nogueira M., Zazá Borges M.A. (2020). Re-Emergence of Yellow Fever in Brazil during 2016–2019: Challenges, Lessons Learned, and Perspectives. Viruses.

[13] Giovanetti M., de Mendonça M.C.L., Fonseca V., Mares-Guia M.A., Fabri A., Xavier J., de Jesus J.G., Gräf T., Dos Santos Rodrigues C.D., Dos Santos C.C. (2019). Yellow Fever Virus Reemergence and Spread in Southeast Brazil, 2016–2019. J. Virol..

[14] Abreu F.V.S., Ferreira-de-Brito A., Azevedo A.S., Linhares J.H.R., de Oliveira Santos V., Hime Miranda E., Neves M.S.A.S., Yousfi L., Ribeiro I.P., Santos A.A.C. (2020). Survey on Non-Human Primates and Mosquitoes Does Not Provide Evidences of Spillover/Spillback between the Urban and Sylvatic Cycles of Yellow Fever and Zika Viruses Following Severe Outbreaks in Southeast Brazil. Viruses.

[15] Lourenço-de-Oliveira R., Failloux A.-B. (2017). Lessons Learned on Zika Virus Vectors. PLoS Negl. Trop. Dis..

[16] Lourenço-de-Oliveira R., Failloux A.-B. (2017). High Risk for Chikungunya Virus to Initiate an Enzootic Sylvatic Cycle in the Tropical Americas. PLoS Negl. Trop. Dis..

[17] Lowe R., Lee S., Martins Lana R., Torres Codeço C., Castro M.C., Pascual M. (2020). Emerging Arboviruses in the Urbanized Amazon Rainforest. BMJ.

[18] Enslen A.W., Lima Neto A.S., Castro M.C. (2020). Infestation Measured by Aedes Aegypti Larval Surveys as an Indication of Future Dengue Epidemics: An Evaluation for Brazil. Trans. R. Soc. Trop. Med. Hyg..

[19] Kilpatrick A.M. (2011). Globalization, Land Use, and the Invasion of West Nile Virus. Science.

[20] Hazin A.N., Poretti A., Di Cavalcanti Souza Cruz D., Tenorio M., van der Linden A., Pena L.J., Brito C., Gil L.H.V., de Barros Miranda-Filho D., Marques E.T.A. (2016). Computed Tomographic Findings in Microcephaly Associated with Zika Virus. N. Engl. J. Med..

[21] Gratz N.G. (2004). Critical Review of the Vector Status of Aedes Albopictus. Med. Vet. Entomol..

[22] Luo L., Li X., Xiao X., Xu Y., Huang M., Yang Z. (2015). Identification of *Aedes Albopictus* Larval Index Thresholds in the Transmission of Dengue in Guangzhou, China. J. Vector Ecol..

[23] Carneiro M.A.F., Reis B.C.A.A., Gehrke F.S., Domingues J.N., Sá N., Paixão S., Figueiredo J., Ferreira A., Almeida C., Machi A. (2017). Environmental Factors Can Influence Dengue Reported Cases. Rev. Assoc. Med. Bras. 1992.

[24] Pereira-dos-Santos T., Roiz D., Lourenço-de-Oliveira R., Paupy C. (2020). A Systematic Review: Is Aedes Albopictus an Efficient Bridge Vector for Zoonotic Arboviruses?. Pathogens.

[25] Kraemer M.U.G., Reiner R.C., Brady O.J., Messina J.P., Gilbert M., Pigott D.M., Yi D., Johnson K., Earl L., Marczak L.B. (2019). Past and Future Spread of the Arbovirus Vectors Aedes Aegypti and Aedes Albopictus. Nat. Microbiol..

[26] Patel P., Landt O., Kaiser M., Faye O., Koppe T., Lass U., Sall A.A., Niedrig M. (2013). Development of One-Step Quantitative Reverse Transcription PCR for the Rapid Detection of Flaviviruses. Virol. J..

[27] Eshoo M.W., Whitehouse C.A., Zoll S.T., Massire C., Pennella T.-T.D., Blyn L.B., Sampath R., Hall T.A., Ecker J.A., Desai A. (2007). Direct Broad-Range Detection of Alphaviruses in Mosquito Extracts. Virology.

[28] Molina J.S.T.O., Carmo A.M.S., Pereira G.L., Andrade L.A., Jordão F.T., Suzuki R.B., Oliveira L.P.R., Cabral A.D., Sperança M.A., Aparecida Sperança M. (2020). Novel Single Hematophagous Insect RNA Detection Method Supports Its Use as Sentinels to Survey Flaviviruses Circulation. Dengue Fever in a One Health Perspective.

[29] Benson D.A., Cavanaugh M., Clark K., Karsch-Mizrachi I., Lipman D.J., Ostell J., Sayers E.W. (2013). GenBank. Nucleic Acids Res..

[30] Mount D.W. (2007). Using the Basic Local Alignment Search Tool (BLAST). Cold Spring Harb. Protoc..

[31] Hill S.C., de Souza R., Thézé J., Claro I., Aguiar R.S., Abade L., Santos F.C.P., Cunha M.S., Nogueira J.S., Salles F.C.S. (2020). Genomic Surveillance of Yellow Fever Virus Epizootic in São Paulo, Brazil, 2016–2018. PLoS Pathog..

[32] Grubaugh N.D., Faria N.R., Andersen K.G., Pybus O.G. (2018). Genomic Insights into Zika Virus Emergence and Spread. Cell.

[33] Lima A.C., Galardo A.K.R., Volmir M.Z., Gomes M., Roux E. (2014). Indicadores Entomológicos Do Risco de Transmissão da Malária Na Fronteira Guiana-Brasileira. ResearchGate.

[34] Forattini O.P. (2002). Culicidologia Médica.

[35] Santos D., Ribeiro G.C., Cabral A.D., Sperança M.A. (2018). A Non-Destructive Enzymatic Method to Extract DNA from Arthropod Specimens: Implications for Morphological and Molecular Studies. PLoS ONE.

[36] Lanciotti R.S., Calisher C.H., Gubler D.J., Chang G.J., Vorndam A.V. (1992). Rapid Detection and Typing of Dengue Viruses from Clinical Samples by Using Reverse Transcriptase-Polymerase Chain Reaction. J. Clin. Microbiol..

[37] Pfeffer M., Linssen B., Parker M.D., Kinney R.M. (2002). Specific Detection of Chikungunya Virus Using a RT-PCR/Nested PCR Combination. J. Vet. Med. Ser. B.

[38] De Morais Bronzoni R.V., Baleotti F.G., Ribeiro Nogueira R.M., Nunes M., Moraes Figueiredo L.T. (2005). Duplex Reverse Transcription-PCR Followed by Nested PCR Assays for Detection and Identification of Brazilian Alphaviruses and Flaviviruses. J. Clin. Microbiol..

[39] Scaramozzino N., Crance J.-M., Jouan A., DeBriel D.A., Stoll F., Garin D. (2001). Comparison of Flavivirus Universal Primer Pairs and Development of a Rapid, Highly Sensitive Heminested Reverse Transcription-PCR Assay for Detection of Flaviviruses Targeted to a Conserved Region of the NS5 Gene Sequences. J. Clin. Microbiol..

[40] Shi C., Beller L., Deboutte W., Yinda K.C., Delang L., Vega-Rúa A., Failloux A.-B., Matthijnssens J. (2019). Stable Distinct Core Eukaryotic Viromes in Different Mosquito Species from Guadeloupe, Using Single Mosquito Viral Metagenomics. Microbiome.

[41] Schultz M.J., Frydman H.M., Connor J.H. (2018). Dual Insect Specific Virus Infection Limits Arbovirus Replication in Aedes Mosquito Cells. Virology.

[42] Marklewitz M., Zirkel F., Kurth A., Drosten C., Junglen S. (2015). Evolutionary and Phenotypic Analysis of Live Virus Isolates Suggests Arthropod Origin of a Pathogenic RNA Virus Family. Proc. Natl. Acad. Sci. USA.

[43] Medeiros A.S., Costa D.M.P., Branco M.S.D., Sousa D.M.C., Monteiro J.D., Galvão S.P.M., Azevedo P.R.M., Fernandes J.V., Jeronimo S.M.B., Araújo J.M.G. (2018). Dengue Virus in Aedes Aegypti and Aedes Albopictus in Urban Areas in the State of Rio Grande Do Norte, Brazil: Importance of Virological and Entomological Surveillance. PLoS ONE.

[44] Barbosa P.P., Guedes D.R.D., Melo-Santos M.A.V., Cordeiro M.T., Acioli R.V., Batista C.A.V., Gonçalves L.S.M., Souza M.F.M., Araújo Y.V., Magalhães F.J.R. (2016). Vector Surveillance for Dengue Virus Detection in the Archipelago of Fernando de Noronha, Brazil. J. Med. Entomol..

[45] Pérez-Pérez J., Sanabria W.H., Restrepo C., Rojo R., Henao E., Triana O., Mejía A.M., Castaño S.M., Rúa-Uribe G.L. (2017). Virological Surveillance of *Aedes* (*Stegomyia*) *Aegypti* and *Aedes* (*Stegomyia*) *Albopictus* as Support for Decision Making for Dengue Control in Medellín. Biomedica.

[46] Chetry S., Patgiri S.J., Bhattacharyya D.R., Dutta P., Kumar N.P. (2020). Incrimination of Aedes Aegypti and Aedes Albopictus as Vectors of Dengue Virus Serotypes 1, 2 and 3 from Four States of Northeast India. Access Microbiol..

[47] Mulyatno K.C., Kotaki T., Yotopranoto S., Rohmah E.A., Churotin S., Sucipto T.H., Amarullah I.H., Wardhani P., Soegijanto S., Kameoka M. (2018). Detection and Serotyping of Dengue Viruses in *Aedes Aegypti* and *Aedes Albopictus* (Diptera: Culicidae) Collected in Surabaya, Indonesia from 2008 to 2015. Jpn. J. Infect. Dis..

[48] (2020). Boletim Epidemiológico do Município de São Paulo (16 de Novembro). https://www.Prefeitura.Sp.Gov.Br/Cidade/Secretarias/Upload/Saude/BoletimArbo_SE47_16nov.Pdf.

[49] Bai Z., Liu L., Tu Z., Yao L., Liu J., Xu B., Tang B., Liu J., Wan Y., Fang M. (2008). Real-Time PCR for Detecting Circulating Dengue Virus in the Guangdong Province of China in 2006. J. Med. Microbiol..

[50] Chakravarti A., Chauhan M., Banerjee S., Roy P. (2016). Comparison of Multiplex RT-PCR and Real-Time HybProbe Assay for Serotyping of Dengue Virus Using Reference Strains and Clinical Samples from India. Indian J. Pathol. Microbiol..

[51] Gurukumar K.R., Priyadarshini D., Patil J.A., Bhagat A., Singh A., Shah P.S., Cecilia D. (2009). Development of Real Time PCR for Detection and Quantitation of Dengue Viruses. Virol. J..

