# Supplementary material for: Dengue-2 and Guadeloupe Mosquito Virus RNA Detected in Aedes (Stegomyia) spp. Collected in a Vehicle Impound Yard in Santo André, SP, Brazil
Source: Insects. 2021 Mar 16;12(3):248. doi: 10.3390/insects12030248 (PMC8001461; doi:10.3390/insects12030248)
Supplement: Supplementary file 1 [file insects-12-00248-s001.pdf]

insects

MDPI

Table S1. Oligonucleotides and target regions in endpoint PCR assay.

Name	5′-3′	Amplicon	Reference
cFD2-Flavi250	GTGTCCCAGCCGGCGGTGTCATCAGC	220-250bp	[1]
MAMD-Flavi250	AACATGATGGGRAARAGRGARAA	NS5	
Chik1-E2F	TAATGCTGAACTCGGGGACC	427bp chik1, chik4	[2]
nChik2-E2F	GATCAGGTTAACCGTGCCGACT	Nested 172bp nchik2, nchik3	
nChik3-E2R	CACTGACACAACTACCACAGTCA	E2 protein	
Chik4-E2R	ACCTGCCACACCCACCATCGAC		
M2W	YAGAGCDTTTTCGCAYSTRGCHW	434bp	[3]
cM3W	ACATRAANKGNGTNGTRTCRAANCCDAYCC	Nested 270bp	

Table S2. Oligonucleotides, probes, and targets in RT-qPCR assay.

Name	5′- 3′	Target (bp)
**Dengue 1** NS2A	Forward: TGTTCGCTGTTGGGCTATCA<br>Reverse: CCTAGCTCCTCCAAAGAGTTT<br>Probe: FAM- ACGATTGGATTAAGCCTGGTGGCA	116
**Dengue 2** NS2B	Forward: GGATGGTGAGCATTTTGGCC<br>Reverse: GGCTCTCTCCAGTTCCAAATC<br>Probe: HEX- GGCTCTCTCCAGTTCCAAATC	134
**Dengue 3** NS2A	Forward: TGGGTGTCTTGTGTTTGGCAATC<br>Reverse: GTGCGTCATGTCTCTCCATGTT<br>Probe: FAM-TTGTGCTCCTTCTCTCAGGGCAA	137
**Dengue 4** 3′UTR	Forward: CGCGTGGCATATTGGACTAC<br>Reverse: GTGCGTCATGTCTCTCCATGTT<br>Probe: HEX-CCATCACCAACGAAACGCAGCAAA	125
**Yellow Fever**	Forward: TGCCATGCCACCCTAACTTA<br>Reverse: CAACCTCTAGCGGCTATGCTA<br>Probe: Cy3-TGGAACCAACTAGGGTTGTTAACTGGG	116
**Zika** GP1	Forward: CTGGCATCATGAAGAATCCCG<br>Reverse: CACTTGTCCCATCTTCTTCTCC<br>Probe: HEX-TGGAATAGTGGTGACTGACATTGACACA	98
**Chikungunya** E2	Forward: CGTAACAGTGATCCCGAAC<br>Reverse: CAGTAGTTCCATCTCCAATACC<br>Probe: Cy3-ACGGTGGGAGTACCGTATAAGACTCT	94

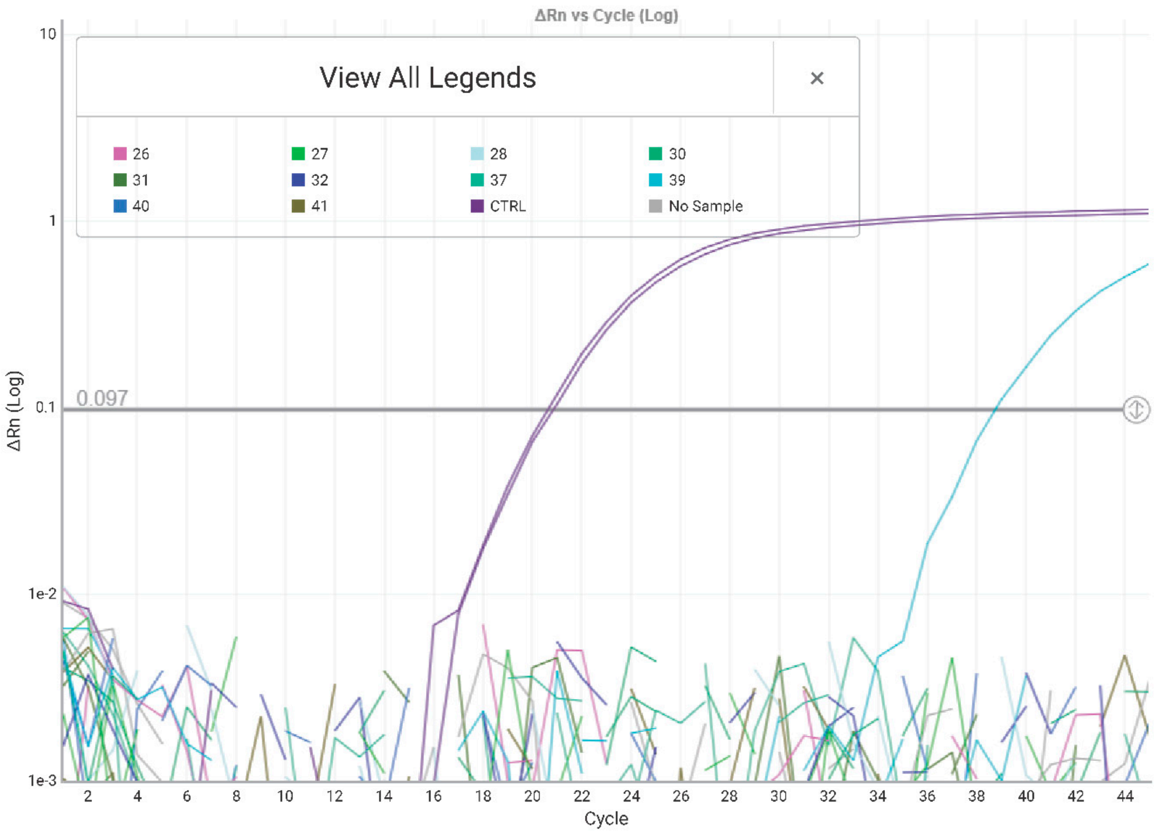

Figure S1. Reaction curve of *Ae. albopictus* female specimen (39) in amplification plot in RT-qPCR.

References

1. Scaramozzino, N.; Crance, J.-M.; Jouan, A.; DeBriel, D.A.; Stoll, F.; Garin, D. Comparison of Flavivirus Universal Primer Pairs and Development of a Rapid, Highly Sensitive Heminested Reverse Transcription-PCR Assay for Detection of Flaviviruses Targeted to a Conserved Region of the NS5 Gene Sequences. *J. Clin. Microbiol.* **2001**, *39*, 1922–1927, doi:10.1128/JCM.39.5.1922-1927.2001.
2. Pfeffer, M.; Linssen, B.; Parker, M.D.; Kinney, R.M. Specific Detection of Chikungunya Virus Using a RT-PCR/Nested PCR Combination. *J. Vet. Med. Series. B* **2002**, *49*, 49–54, doi:10.1046/j.1439-0450.2002.00535.x.
3. de Morais Bronzoni, R.V.; Baleotti, F.G.; Ribeiro Nogueira, R.M.; Nunes, M.; Moraes Figueiredo, L.T. Duplex reverse transcription-PCR followed by nested PCR assays for detection and identification of Brazilian alphaviruses and flaviviruses. *J. Clin. Microbiol.* **2005**, *43*, 696–702, doi:10.1128/JCM.43.2.696-702.2005.
